# Assessment of Post-treatment Quality of Life in Breast Cancer Patients Using the EORTC QLQ-C30: A Prospective Observational Study

**DOI:** 10.7759/cureus.97801

**Published:** 2025-11-25

**Authors:** Simranpreet Singh Kahlon, Divya Dahiya, Ruchika Kumari, Maninder Deep Kaur, R.N. Naga Santhosh Irrinki, Satish Subbiah Nagaraj

**Affiliations:** 1 Urology, Rabindranath Tagore Medical College, Udaipur, IND; 2 General Surgery, Postgraduate Institute of Medical Education and Research, Chandigarh, IND; 3 General Surgery, Pandit Bhagwat Dayal Sharma Post Graduate Institute of Medical Sciences, Rohtak, IND; 4 Nursing, Postgraduate Institute of Medical Education and Research, Chandigarh, IND

**Keywords:** breast cancer, eortc qlq-c30, global health score, indian women, quality of life

## Abstract

Background: Breast cancer is the most common cancer among Indian women. Although advances in treatment have improved survival rates, many patients continue to face physical and emotional difficulties that impact quality of life (QOL) even after completing therapy. This study aimed to assess the QOL in breast-cancer patients three months after treatment using the European Organization for Research and Treatment of Cancer Quality of Life Questionnaire (EORTC QLQ-C30).

Methods: A prospective observational study was conducted on 100 breast-cancer patients at Postgraduate Institute of Medical Education and Research (PGIMER), Chandigarh. Patients were assessed three months after treatment completion. Sociodemographic and clinical details were recorded. QOL was measured using the EORTC QLQ-C30. Descriptive statistics were performed. Parametric tests were applied for continuous variables with normal distribution; otherwise, non-parametric tests were used. Pearson’s and Spearman’s correlation coefficients, along with multiple regression modelling, were used wherever applicable.

Results: Mean age of study population was 51.02±12.16 years. Mean Global Health Score (GHS) QOL score was 55.17± 13.75, which was lower than the EORTC reference value of 61.8 ±24.6. Among functional scales, the best score was for emotional functioning (EF) (91.00 ± 11.22) while social functioning (SF) scored the lowest (74.33± 18.26). Pain, insomnia, and fatigue were the most worrisome symptoms. Elder age negatively affected GHS, physical functioning (PF), and cognitive functioning (CF). Being married had a significant positive impact on GHS, PF, and role functioning (RF). Living in a nuclear family and having an occupation have a significant positive impact on GHS.

Conclusions: There were many factors which were related to lower QOL, including age, marital status, occupation status, and type of family. This study highlights the women at risk of a poorer QOL after breast-cancer treatment in Indian society.

## Introduction

Breast cancer remains the most frequently diagnosed malignancy among women worldwide, accounting for approximately 2.3 million new cases and 670,000 deaths in 2022 [1,2]. It is the leading cause of cancer-related morbidity among females in both developed and developing regions. In India, breast cancer has emerged as the most common cancer among women, with an estimated 192,020 new cases reported in 2022, representing about 14% of all new cancer diagnoses in the country [3]. The age-standardized incidence rate (ASIR) for female breast cancer in India is approximately 26.6 per 100,000 women, according to GLOBOCAN 2022 estimates [4]. 

Advancements in breast-cancer management, including the introduction of breast-conserving surgeries, targeted therapies, improved chemotherapeutic regimens, and adjuvant radiotherapy, have significantly improved survival rates [5,6]. However, various treatment-related morbidities persist in breast-cancer patients even after the completion of therapy [7].

Patients often experience persistent physical symptoms related to surgical procedures, chemotherapy, and radiotherapy [8]. In addition to physical symptoms, many patients also experience significant psychological and emotional challenges, including anxiety, depression, fear of recurrence, and reduced social well-being, which further impact their recovery. Collectively, these side effects contribute to a decline in the quality of life (QOL) of breast-cancer patients after treatment [9-11]. 

Beyond curative intent, cancer treatment now emphasizes enhancing patients overall QOL. It encompasses physical, emotional, social, and cognitive well-being. Various tools are available to assess QOL in cancer survivors. Among them, the European Organization for Research and Treatment of Cancer Quality of Life Questionnaire (EORTC QLQ-C30) stands out as a validated, widely used instrument specifically designed for cancer patients. It evaluates functional domains and symptom burden, offering a comprehensive overview of the patients’ post-treatment state [12].

Despite growing interest in survivorship care globally, there is limited literature from India assessing QOL outcomes after breast-cancer treatment. Some studies from West India, particularly those conducted at the Tata Memorial Centre, Mumbai, have evaluated QOL in breast-cancer patients using the EORTC QLQ-C30 and BR-23 questionnaires, demonstrating the tool’s reliability and cultural relevance in the Indian setting [13,14]. However, such research remains scarce in other regions of the country. Unique sociocultural factors, such as extended family structures, gender roles, limited access to mental health resources, and lower health literacy, may further influence recovery and psychosocial adaptation in Indian women. This study aims to evaluate QOL among Indian breast-cancer patients three months post treatment using the EORTC QLQ-C30 and to identify key sociodemographic and clinical predictors that influence QOL in Indian women.

This study was previously presented as a poster at the European Breast Cancer Conference (EBCC), published in the European Journal of Cancer Supplement 2024. It was also presented at the Indian National Conference ABSICON 2023 in Ahmedabad, India.

## Materials and methods

This prospective observational study was conducted at the Department of General Surgery in Postgraduate Institute of Medical Education and Research (PGIMER), Chandigarh. Ethical clearance was obtained from the institutional ethics committee. A total of 100 biopsy-proven breast-cancer patients who had completed their treatment were included. Patients aged 18 years or older who provided informed consent were eligible for the study. Those with metastatic disease, psychiatric illness, or cognitive impairment that could interfere with questionnaire responses were excluded.

All participants were assessed three months after treatment completion. Sociodemographic and clinical details, including age, marital status, education, occupation, locality, family type, BMI, stage at diagnosis, and treatment received, were collected using a structured Proforma.

QOL was evaluated using the EORTC QLQ-C30, which assesses five functional domains (physical, role, emotional, cognitive, and social functioning (PF, RF, EF, CF, and SF)), nine symptom scales (fatigue, nausea and vomiting, pain, dyspnea, insomnia, appetite loss, constipation, diarrhea, and financial difficulties), and a global health status (GHS) score. The questionnaire was administered in either Hindi or Punjabi, based on the participant’s language preference. The use of both language versions was authorized by the EORTC.

Scoring followed the EORTC QLQ-C30 manual, transforming raw scores into a 0-100 scale. Higher scores on the functional and global health scales reflected better QOL, while higher scores on the symptom scales indicated greater symptom burden.

Formal permission to use the EORTC QLQ-C30 was obtained via e-mail (Request ID: 76979) from the EORTC Quality of Life Group, prior to commencement of the study. 

Statistical analysis

Descriptive statistics were used for sociodemographic, clinical, treatment-related, and QOL variables. Parametric tests were applied for continuous variables with normal distribution; otherwise, non-parametric tests were used. Categorical variables were expressed as frequencies and percentages. Correlations between continuous variables and QOL were assessed using Pearson’s correlation coefficient. For categorical variables, Spearman’s correlation coefficient was applied. Multiple regression analysis was performed to identify predictors of QOL based on factors found significant in correlation analysis and those deemed clinically relevant. A p-value of < 0.05 was considered statistically significant. 

## Results

The mean age of the participants was 48.5 ± 10.2 years, with 54 (54%) aged over 50 years. Most of the participants were married (89, 89%). Urban and rural participants were nearly equally distributed. Most participants belonged to nuclear families, and the majority were homemakers, as shown in Table 1.

**Table 1:** Demographic characteristics of the participants

Variable	Category	Frequency (n=100)
Age (mean ± SD)	48.5 ± 10.2	-
	Up to 50 years	46
	More than 50 years	54
Marital Status	Married	89
	Widow	9
	Unmarried	1
	Divorced	1
Education Level	≥12th Standard	42
	10th Standard	12
	Middle Level	6
	No Formal Education	40
Locality	Urban	51
	Rural	49
Family Type	Joint	32
	Nuclear	68
Occupation	Working	18
	Homemaker	82

Among study participants, 61 (61%) had early-stage and 39 (39%) had advanced-stage breast cancer. Clinical and treatment details are presented in Table 2.

**Table 2:** Clinical characteristics of the study participants NACT: Neoadjuvant chemotherapy

Variable	Category	Frequency (n=100)
Cancer Stage	Early Stage (I & II)	61
	Advanced Stage (III)	39
Treatment	Upfront Surgery	42
	NACT → Surgery	58
Radiotherapy	Received	85
	Not Received	15
BMI (kg/m²)	≤25	65
	>25	35
Menopausal Status	Premenopausal	44
	Postmenopausal	56

QOL scores

The mean GHS score was 55.17 ± 13.75. EF had the highest mean score (91.00 ± 11.22), while SF was the lowest (74.33 ± 18.26). Among symptoms scale, pain (41.17 ± 22.78), insomnia (22.33 ± 24.18), and fatigue (18.44 ± 16.80) were most worrisome. Detailed QOL scores of all patients are shown in Table 3.

**Table 3:** QOL score of patients EORTC: European Organization for Research and Treatment of Cancer; GHS: Global health score; QOL: Quality of life, PF: Physical functioning; RF: Role functioning; EF: Emotional functioning; CF: Cognitive functioning; SF: Social functioning

EORTC variable	Mean ± SD
GHS	55.17 ± 13.75
Functioning Scale	
PF	75.87 ± 11.28
RF	81.17 ± 17.67
EF	91.00 ± 11.22
CF	84.83 ± 18.97
SF	74.33 ± 18.26
Symptom Scale	
Fatigue	18.44 ± 16.80
Pain	41.17 ± 22.78
Nausea/Vomiting	07.00 ± 12.12
Insomnia	22.33 ± 24.18
Appetite Loss	06.33 ± 15.49
Constipation	03.00 ± 10.69
Diarrhea	06.00 ± 15.26
Functional Difficulties	12.33 ± 18.13

Sociodemographic and clinical variable with EORTC-QLQ-C30 functional scales

Patients aged ≤50 years demonstrated significantly better GHS, as well as superior PF, RF, and CF compared to those aged >50 years (p<0.05). Married and working women also showed higher global health and PF scores. Nuclear family type was associated with better global health (p=0.011). No significant differences were observed across BMI, menopausal status, radiotherapy, or disease stage. Detailed correlation between sociodemographic and clinical variables with functional scale is shown in Tables 4 and 5, respectively.

**Table 4:** Correlation between sociodemographic factors and QOL domains PF: Physical functioning; RF: Role functioning; CF: Cognitive functioning; EF: Emotional functioning; SF: Social functioning; GHS: Global health status; QOL: Quality of life

Domains	GHS	PF	RF	EF	CF	SF
Age	r=-0.526	r=-0.317	r=-0.261	r=0.193	r=-0.364	r=0.048
	p<0.001	p=0.001	p=0.009	p=0.055	p<0.001	p=0.637
Marital Status (Married)	r=0.219	r=0.262	r=0.263	r=0.019	r=0.083	r=-0.013
	p=0.028	p=0.009	p=0.008	p=0.848	p=0.412	p=0.896
Nuclear Family	r=0.254	r=0.185	r=0.104	r=-0.135	r=0.042	r=-0.071
	p=0.011	p=0.066	p=0.304	p=0.182	p=0.677	p=0.482
Locality (Rural)	r=-0.010	r=0.076	r=-0.075	r=0.113	r=0.146	r=0.055
	p=0.921	p=0.454	p=0.456	p=0.263	p=0.147	p=0.585
Occupation (Working)	r=0.236	r=0.126	r=0.057	r=-0.101	r=0.179	r=-0.021
	p=0.018	p=0.212	p=0.571	p=0.319	p=0.075	p=0.836

**Table 5:** Correlation between clinical variables and QOL domains PF: Physical functioning; RF: Role functioning; CF: Cognitive functioning; EF: Emotional functioning; SF: Social functioning; GHS: Global health status; QOL: Quality of life

Domains	GHS	PF	RF	EF	CF	SF
BMI	r=-0.106	r=-0.354	r=-0.025	r=-0.029	r=-0.643	r=-0.356
	p=0.074	p=0.098	p=0.823	p=0.763	p=0.065	p=0.451
Time Since Diagnosis to Surgery	r=0.067	r=-0.037	r=0.040	r=-0.047	r=-0.063	r=-0.092
	p=0.508	p=0.715	p=0.694	p=0.643	p=0.531	p=0.363
Stage (Late)	r=0.108	r=0.051	r=0.177	r=-0.116	r=0.042	r=0.025
	p=0.287	p=0.617	p=0.077	p=0.249	p=0.680	p=0.808
Radiotherapy Received	r=-0.018	r=0.022	r=0.005	r=-0.117	r=-0.168	r=-0.124
	p=0.858	p=0.830	p=0.960	p=0.248	p=0.095	p=0.221

## Discussion

In this study, the mean GHS score was 55.17 ± 13.75, which is lower than the EORTC reference value of 61.8 and considerably lower than the mean score of 76.9 ± 19.2, reported in a large pooled analysis by Mierzynska et al. in the western population with early breast-cancer patients [15]. These findings reflect the multifaceted challenges faced by Indian patients, including treatment-related fatigue, limited access to post-treatment rehabilitation, financial constraints, and lack of psychosocial support services [16].

Increasing age in the current study was associated with significantly lower GHS and reduced scores in PF, RF, CF, and EF. Similar findings have been reported in Indian literature, such as by Ghosh et al., who observed poorer QOL outcomes in elderly breast-cancer patients due to reduced resilience and higher treatment burden [17]. Interestingly, Sammarco found that breast-cancer survivors above 50 years of age in Western populations reported better psychological and spiritual well-being despite comparable physical decline, suggesting stronger emotional adaptation with age [18]. Such contrast may be due to differences in healthcare access, social support systems, and cultural expectations regarding aging and illness. In this study, lower QOL in older patients may be attributed to age-related comorbidities, reduced physiological reserve, and increased psychological stress-all of which can negatively impact QOL following treatment, similar to other studies [19,20]. 

In this study, both marital status and family structure influenced post-treatment QOL. Married women showed significantly better global health and functional scores compared to their unmarried or widowed counterparts, consistent with findings by Konieczny et al., who reported that married breast-cancer patients exhibited better CF, greater optimism about the future, and lower symptom intensity [21]. In Indian society, spouses often provide critical emotional, logistical, and financial support during illness, enhancing overall recovery.

Patients living in nuclear families also reported better GHS, possibly due to greater autonomy and reduced household burden. In contrast, women in joint families often encounter traditional gender role expectations and multiple caregiving responsibilities within the extended family, which can influence various aspects of their QOL [22].

Employment status significantly influenced QOL. Working women had better global health scores. This finding is corroborated by Timperi et al., who emphasized that employment post treatment enhances emotional resilience and provides a sense of purpose [23]. A recent Indian study by Kumari et al. also reported that having an occupation contributes to better mental and physical well-being among breast-cancer patients [24]. 

Among symptoms, pain, insomnia, and fatigue were the most commonly reported issues, consistent with the findings of Kaur et al., who observed persistent fatigue even after chemotherapy [8]. Similarly, the Asian meta-analysis by Chen et al. described a comparable symptom cluster in breast-cancer patients across Asia, where fatigue, pain, and insomnia frequently co-occur and significantly reduce overall QOL. The study also noted that distress due to hair loss and mood disturbances often accompany these physical symptoms, highlighting the multifactorial nature of post-treatment distress in Asian women. These findings emphasize the need for targeted management strategies [25].

This study highlights specific groups of post treatment breast-cancer patients who require focused care. Elderly patients required age specific rehabilitation and their families should be guided to support them better. Single women, being more vulnerable to poor emotional health, should be offered psychological counseling, enrolled in peer support groups or referred to psychotherapy. Patients from joint family settings may benefit from family-centered counseling to foster a more understanding home environment. Lastly, given the clear link between employment and better QOL, women should be actively encouraged to return to work or pursue any form of occupation, promoting both financial autonomy and self-worth. These culturally sensitive, patient-centered interventions are essential to improving QOL after treatment in Indian breast-cancer patients.

## Conclusions

The finding of this study demonstrates that breast-cancer treatment substantially influences women’s QOL in India. While emotional resilience remains relatively preserved, challenges in PF and SF persist, particularly among older women, homemakers, and those living in joint families. Pain, fatigue, and insomnia were frequently reported, underscoring the need for structured interventions to support recovery. Sociodemographic factors, such as age, marital status, employment, and family structure, significantly influenced outcomes. Education and awareness must extend beyond patients to families and communities, as social context strongly shapes recovery. By integrating symptom-management strategies, psychosocial counseling, and family/community education into post-treatment care, healthcare providers can improve recovery and enhance overall QOL for breast-cancer patients in India.
